# Beyond a Single Marker: An Update on the Comprehensive Evaluation of Right Ventricular Dysfunction in Pulmonary Thromboembolism

**DOI:** 10.3390/life15040665

**Published:** 2025-04-17

**Authors:** Sandu Cucută, Minerva Codruta Badescu, Ștefania-Teodora Duca, Adriana Chetran, Maria-Ruxandra Cepoi, Cosmina-Georgiana Ponor, Amelian Madalin Bobu, Ionela-Lacramioara Serban, Irina-Iuliana Costache-Enache

**Affiliations:** 1 Department of Internal Medicine I, Faculty of Medicine, “Grigore T. Popa” University of Medicine and Pharmacy, 700115 Iasi, Romania; cucuta.sandu@d.umfiasi.ro (S.C.); stefania-teodora.duca@email.umfiasi.ro (Ș.-T.D.); adriana.ion@umfiasi.ro (A.C.); cepoi_maria-ruxandra@d.umfiasi.ro (M.-R.C.); irina.costache@umfiasi.ro (I.-I.C.-E.); 2 Cardiology Clinic, “St. Spiridon” County Emergency Clinical Hospital, 700111 Iasi, Romania; cosminageorgiana-ponor@email.umfiasi.ro (C.-G.P.); amelian.bobu@yahoo.com (A.M.B.); 3 IIIrd Internal Medicine Clinic, “St. Spiridon” County Emergency Clinical Hospital, 700111 Iasi, Romania; 4 Department of Morpho-Functional Sciences II, Discipline of Physiology, Faculty of Medicine, “Grigore T. Popa” University of Medicine and Pharmacy, 700115 Iasi, Romania; ionela.serban@umfiasi.ro

**Keywords:** pulmonary thromboembolism, right ventricular dysfunction, NT-proBNP, H-FABP, GDF-15, hs-cTnI

## Abstract

Pulmonary thromboembolism (PE) is a life-threatening condition that often leads to right ventricular (RV) dysfunction, a key determinant of prognosis and clinical management. Biomarkers play a crucial role in the early detection and risk stratification of RV dysfunction in PE, complementing imaging and hemodynamic assessments. Cardiac troponins, B-type natriuretic peptides, and novel biomarkers, such as heart-type fatty acid-binding protein (H-FABP) and growth differentiation factor-15 (GDF-15), provide valuable insights into myocardial injury, overload, and stress. This article explores the clinical possible significance of these biomarkers, their predictive value, and their potential to guide therapeutic strategies in patients with PE. Understanding the role of biomarkers in RV dysfunction assessment may improve patient outcomes focusing on early intervention and personalized treatment approaches.

## 1. Introduction

Pulmonary thromboembolism (PE) is a life-threatening cardiovascular condition caused by obstruction of the pulmonary arteries due to thrombotic emboli, most commonly originating from deep vein thrombosis (DVT), and is a major cause of morbidity and mortality worldwide [1,2]. The global incidence of PE varies, with estimates ranging from 39 to 115 cases per 100,000 individuals annually [1,2]. The prevalence of venous thromboembolism (VTE), which includes both deep vein thrombosis and pulmonary embolism, has been estimated at approximately 1–2 per 1000 people per year [3]. Mortality rates for PE are significant, particularly in untreated cases, with reported mortality rates as high as 30%. However, due to appropriate anticoagulant treatment and risk stratification, overall mortality has decreased to approximately 2% to 8% in contemporary cohorts [4,5]. Despite advances in diagnosis and management, PE remains a leading cause of sudden cardiovascular disease-related death, emphasizing the need for improved risk stratification tools and treatment approaches [1,2,4].

Right ventricular (RV) dysfunction plays a pivotal role in the pathophysiology of PE, and is a key determinant of clinical outcomes [6,7]. When a pulmonary embolus obstructs the pulmonary arterial circulation, it leads to increased pulmonary vascular resistance, causing RV pressure overload, resulting in RV dilation, impaired systolic function and, ultimately, hemodynamic instability [6,7]. Clinically, RV dysfunction is associated with increased short-term mortality, particularly in intermediate- and high-risk PE cases [6,7]. Methods for diagnosing RV dysfunction include transthoracic echocardiography (TTE), computed tomography pulmonary angiography (CTPA), and cardiac biomarkers [8,9].

Biomarkers have been increasingly used to assess RV dysfunction in PE, as they provide valuable, non-invasive, and easily accessible prognostic information. Common biomarkers reflecting RV wall stress include troponins (cardiac troponin I and T), brain natriuretic peptide (BNP), or its N-terminal precursor proBNP (NT-proBNP). Increased levels are associated with poorer clinical outcomes, including increased mortality and hemodynamic decompensation [10,11,12]. Other emerging biomarkers, such as heart-type fatty acid-binding protein (H-FABP) and growth differentiation factor-15 (GDF-15), have also been studied for their potential to improve risk stratification [10,11,12].

Despite significant advances in the diagnosis and management of PE, including improved imaging techniques and validated prognostic scores such as the Pulmonary Embolism Severity Index (PESI) and European Society of Cardiology (ESC) risk stratification models, a critical need for further refinement of risk assessment tools still remains [13]. The inclusion of novel biomarkers alongside existing clinical and imaging parameters has the potential to increase the accuracy of risk stratification, leading to more personalized therapeutic strategies. This review aims to provide an updated overview of novel biomarkers associated with RV dysfunction in PE, and their potential role in clinical practice to facilitate earlier identification of patients at risk for adverse outcomes, optimize therapeutic approaches and, ultimately, improve survival rates. While advanced imaging modalities such as cardiac magnetic resonance imaging (MRI), 3D echocardiography, and cardiopulmonary exercise testing provide additional insights into RV performance, they are not included in the scope of this review due to their limited accessibility and feasibility in acute care settings. Instead, we aim to highlight the diagnostic and prognostic value of biomarkers as practical, non-invasive tools in clinical decision-making algorithms, particularly in intermediate-risk PE.

## 2. Pathophysiological Overview and Risk Assessment of Right Ventricular Dysfunction in Pulmonary Thromboembolism

### 2.1. Physiology and Pathophysiological Mechanisms of RV Dysfunction in PE

RV dysfunction is a critical complication of pulmonary embolism (PE), characterized by the inability of the RV to maintain adequate output flow due to increased afterload [6,7]. The primary mechanism of RV dysfunction in PE is pressure overload resulting from acute obstruction of the pulmonary arteries, leading to increased pulmonary vascular resistance, RV dilation, and reduced contractility [6,7]. Prolonged pressure overload can lead to ischemia due to increased myocardial oxygen demand and reduced coronary perfusion, further exacerbating myocardial dysfunction [7]. Ultimately, RV dysfunction leads to decreased left ventricular preload, systemic hypotension, and cardiogenic shock in severe cases [14]. Understanding these pathophysiological changes is essential for early identification and management of high-risk patients with PE.

The RV has a unique anatomical structure that differentiates it from the left ventricle (LV) in terms of morphology, function, and hemodynamic workload [15,16,17]. Historically considered less important than the LV, the RV has gained recognition due to its impact on heart failure (HF), pulmonary hypertension (PH), congenital heart disease, and post-cardiac intervention outcomes [18,19]. In contrast to the LV, the RV is structurally distinct, with a thinner wall, crescent shape, and a function tailored to the low-pressure pulmonary circulation [6,20]. The RV consists of three main anatomical components, the inlet, which includes the tricuspid valve and papillary muscles, the trabeculated apex, and the outflow tract (infundibulum), leading to the pulmonary valve [6,20]. The RV has a larger volume, but lower mass, compared to the LV, and relies for ejection mainly on longitudinal shortening rather than radial contraction [6,20]. Additionally, it has a highly compliant capacity, allowing it to adapt to variations in preload with minimal pressure changes. The presence of a highly trabeculated endocardial surface and a moderator band is another distinguishing feature of the RV [6,20]. The RV contraction consists of a coordinated sequence involving the free wall, interventricular septum, and LV [6,20]. The LV plays a supportive role in RV function, as septal contraction contributes significantly to RV ejection [6,20]. Ventriculoarterial coupling is another particularity, which ensures efficient blood flow through the pulmonary circulation with minimal energy expenditure [21,22]. In addition, the lower RV cavity pressures allow myocardial perfusion throughout diastole and systole, in contrast to LV, where perfusion occurs only when cavity pressures are lower than aortic root pressures during diastole [23]. These mechanisms allow the RV to function efficiently under physiologic conditions [23].

**Figure 1:**
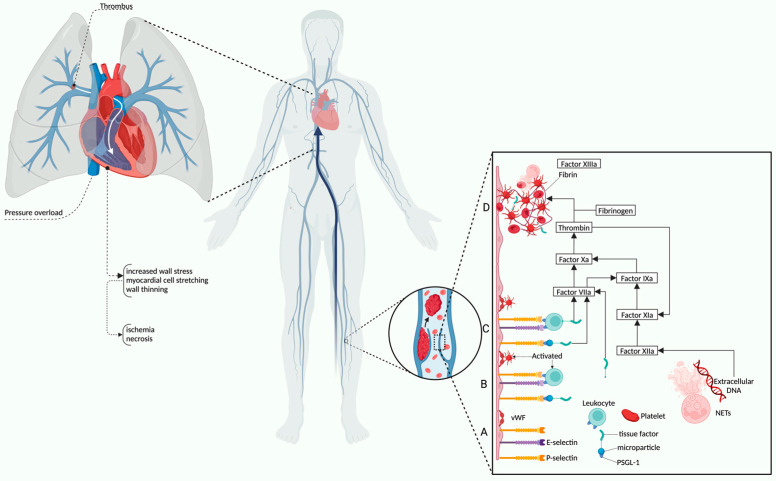
Pathophysiology of right ventricular dysfunction and deep vein thrombosis mechanism. vWF, von Willebrand factor; PSGL-1, P-selectin glycoprotein ligand-1; NETs, neutrophil extracellular traps. A. Endothelial activation; B. Leukocyte activation; C. Coagulation cascade; D. Thrombus formation. Created in BioRender (https://BioRender.com/o16g977, accessed on 16 April 2025).

These structural differences have significant implications in PE, where acute pressure overload can rapidly lead to RV failure due to its limited capacity to adapt to high pressure afterload. A significant increase in RV afterload or a significant decrease in RV contractility, often aggravated by an increased RV preload, can trigger the pathophysiologic mechanisms of RV failure [18,19,21,22]. Three major pathways lead to RV dysfunction. The first is pressure overload that occurs in pulmonary hypertension (PH) and PE. The second is volume overload, seen in valvular regurgitations and congenital heart defects. The last is primary myocardial dysfunction, found in arrhythmogenic cardiomyopathy and ischemic heart disease, where RV contractility is directly affected. [24,25]. The most common cause is DVT, which plays a central role in the pathogenesis of PE, serving as the primary source of thrombi that migrate into the pulmonary circulation. [26,27]. Figure 1 illustrates the multistep pathophysiological mechanisms, starting at the molecular level with endothelial activation and recruitment of leukocytes and platelets. Adhesion molecules, such as von Willebrand factor (vWF), P-selectin, and P-selectin glycoprotein ligand-1 (PSGL-1), are key mediators that promote cellular interactions and progression through the coagulation cascade [26,27]. Tissue factor-bearing microparticles and activated leukocytes contribute to thrombin generation and fibrin clot formation, while neutrophil extracellular traps (NETs) further enhance thrombogenesis and clot stability [26,27]. Once formed, these thrombi can dislodge and embolize in the pulmonary arteries, leading to acute obstruction, increased pulmonary vascular resistance, and triggering the cascade of events leading to RV dysfunction [26,27]. This interplay between systemic inflammation, coagulation, and hemodynamic compromise underlies the clinical severity and complexity of PE.

Each mechanism has distinct clinical and prognostic implications. However, there is also the possibility that all of these mechanisms intertwine [24,25]. PE-induced vasoconstriction, mediated by the release of thromboxane A2 and serotonin, contributes to the initial increase in pulmonary vascular resistance (PVR) and proportional decrease in arterial compliance [16,28,29]. The sudden increase in RVP causes RV dilatation, thereby altering the contractile properties of the myocardium according to the Frank–Starling mechanism [16,28,29]. Once this compensatory capacity is exceeded, maladaptive dilation leads to a subsequent worsening of RV performance and a sudden decrease in RV output [16,28,29]. Dilatation of the RV cavity extends to the tricuspid annulus, producing functional tricuspid regurgitation, increasing preload, and worsening RV distension [16,28,29]. Increased RV pressure and volume lead to an increase in parietal tension, myocyte stretch, and thinning of the RV myocardium [30,31]. Higher parietal stress increases myocardial oxygen demand and reduces myocardial perfusion of the RV, which remains perfused only during diastole [30,31]. This imbalance between increased oxygen demand and reduced myocardial perfusion leads to ischemia, myocyte necrosis, and impaired RV contractility [30,31].

The RV contraction time is prolonged, while neurohumoral activation leads to inotropic and chronotropic stimulation. Together with systemic vasoconstriction, these compensatory mechanisms increase pulmonary artery pressure, improving flow through the obstructed pulmonary vascular bed and thereby temporarily stabilizing systemic blood pressure (BP) [32,33]. The degree of immediate adaptation is limited, as an unconditioned, thin-walled RV is unable to generate a mean pulmonary arterial pressure (PAP) > 40 mmHg [33,34]. Prolongation of RV contraction time leads to leftward bulging of the interventricular septum. The right bundle branch block development further amplifies the asymmetry of ventricular contraction [34,35]. As a result, LV filling is impeded in early diastole, that may lead to a reduction in cardiac output and contribute to systemic hypotension and hemodynamic instability [34,35].

These processes, particularly ischemia, myocardial stretch, and myocyte injury, drive the release of circulating biomarkers that reflect the severity of RV strain. High-sensitivity cardiac troponin I (hs-cTnI) is released in response to ischemic injury of the RV myocardium, and it serves as a highly sensitive but non-specific indicator of myocardial damage [14,36,37]. NT-proBNP is secreted in response to ventricular wall stretch and reflects pressure overload. H-FABP, due to its rapid release following injury, can detect myocardial stress even earlier than troponins. GDF-15, a stress-responsive cytokine, provides prognostic information related to both acute and long-term outcomes [14,36,37]. Although limited in specificity, these biomarkers are valuable in identifying high-risk patients and guiding therapeutic decisions when used in conjunction with clinical and imaging findings [14,36,37].

### 2.2. Clinical Assessment and Risk Stratification of RV Dysfunction

The 2019 European Society of Cardiology (ESC) guidelines emphasize the prognostic importance of RV dysfunction and myocardial injury in stratifying patients with acute PE [13]. Hemodynamic instability remains the defining criteria for high-risk PE, whereas intermediate-risk classification requires evidence of RV dysfunction and/or elevated cardiac biomarkers in normotensive patients [13].

Clinically, nonspecific symptoms such as hypotension, tachycardia, dyspnea, and signs of right heart failure, including jugular venous distension and peripheral edema, suggest RV dysfunction [38,39]. Diagnosis of RV dysfunction is difficult because of the wide range of clinical presentations with limited availability or feasibility for imaging techniques, especially in an acute scenario [40,41]. The particular benefit of biomarkers is that they can provide biochemical evidence of RV strain and myocardial injury, thus allowing earlier diagnosis and stratification [42]. Given the above pathophysiologic considerations, acute RV dysfunction, defined as a rapidly progressive syndrome with systemic congestion resulting from deficient RV filling and/or reduced RV blood flow, is a critical determinant of clinical severity and prognosis in acute PE [13,43]. Establishing the risk of patients with PE is mandatory to determine the appropriate therapeutic approach [13,43]. Initial risk stratification is based on the clinical signs and symptoms of hemodynamic instability, which indicate an increased risk of early death [13,43]. In patients with PE presenting without hemodynamic instability, further risk stratification requires the evaluation of two sets of prognostic criteria. This involves the use of clinical, imaging, and laboratory indicators of PE severity, mainly related to the presence of RV dysfunction and of comorbidities and other aggravating conditions that may adversely affect early prognosis [13,43].

Patients without shock or hypotension are stratified into intermediate-high and intermediate-low risk categories based on imaging and biomarker findings [13]. Intermediate-high risk is defined by the presence of both RV dysfunction (seen on TTE or CTPA) and elevated biomarkers (e.g., hs-cTnI, NT-proBNP) [13]. Those with only one positive parameter fall into the intermediate-low risk group [13]. Clinical scoring systems further assist in risk stratification.

The PESI, in its original or simplified form (sPESI), is the most widely validated and used clinical score to date, as it integrates core indicators of the severity of the acute PE episode, including the patient’s aggravating conditions and comorbidities [13]. It predicts 30-day mortality based on clinical variables such as age, heart rate, systolic blood pressure, and oxygen saturation [13]. Another clinical tool is the Bova Score, which assesses the risk of complications in intermediate-risk PE patients based on systolic blood pressure, cardiac biomarkers (troponins, BNP), and RV dysfunction [44].

In general, a class III PESI or sPESI of 0 is a reliable predictor of low-risk PE [13]. Patients in the intermediate-risk group presenting with both evidence of RV dysfunction (on echocardiography or CTPA) and elevated circulating cardiac biomarker values (especially a positive cardiac troponin test) in addition to clinical parameters, are classified in the intermediate-high risk category [13]. Patients with normal RV on echocardiography or CTPA and/or normal cardiac biomarker values belong to the intermediate-low risk category [13]. A meta-analysis by Barco et al. which included 21 cohort studies with a total of 3295 patients in the low-risk category with a PESI III or sPESI 0 score, found that 34% of patients had evidence of RV dysfunction on echocardiography or CTPA [45]. Despite these tools, up to one-third of low-risk patients (by PESI/sPESI) show evidence of RV dysfunction on imaging, indicating that clinical scores alone may underestimate disease severity [13]. As such, echocardiography remains essential in assessing RV size and function [13]. Although the gold standard for non-invasive measurements of RV size and function is cardiac magnetic resonance imaging, it is time-consuming, often not feasible in daily clinical practice nor cost-effective [46].

Therefore, assessment of RV dysfunction by echocardiography is essential in the management of patients, especially in the setting of acute PE, and is the first line of imaging assessment used in clinical practice [25,46]. However, echocardiographic assessment of the RV is difficult due to its complex anatomy, unfavorable position in the thoracic cavity, and trabeculated myocardium that prevents clear tracing of endocardial boundaries. Thus, multimodal imaging assessment is indicated [47].

The main echocardiographic parameters used to evaluate RV dysfunction are RV dilatation, RV ejection fraction, myocardial performance index (MPI-Tei index), tricuspid annular plane systolic excursion (TAPSE), Doppler tissue imaging (DTI)-derived tricuspid lateral annular systolic velocity (S’t), and estimated pulmonary arterial pressure (PAPs). Continuous Doppler (CW) assessment of tricuspid and pulmonary regurgitant velocities allows the estimation of pulmonary artery pressures and is an essential part of RV assessment [25,46,47]. Longitudinal strain (RVLS) is a dimensionless measure of myocardial function, describing the myocardial deformation occurring during the cardiac cycle and it is currently the standard method for RV assessment in clinical practice [48].

Unlike the LV, which shortens relatively symmetrically in the transverse and longitudinal plane, the orientation of RV muscle fibers constrains contraction to occur predominantly along the longitudinal plane [48]. As a result, the systolic displacement of the tricuspid annulus toward the RV apex (longitudinal plane), termed TAPSE, is closely correlated with RV ejection fraction [48]. TAPSE represents the maximum apexward displacement of the tricuspid annulus in systole, and it is a global indicator of RV longitudinal contraction function [25,46,47]. The normal value for TAPSE is above 16 mm [25,46,47]. However, in some cases of severe pulmonary arterial hypertension, RV systolic function may be significantly reduced despite a pseudo-normalized TAPSE. On the contrary, RV performance may be preserved in the context of a reduced TAPSE, as frequently observed after cardiac surgery [25,46,47]. However, TAPSE is the most commonly used index for the assessment of RV systolic function because it is easy to obtain, reproducible, and demonstrates both diagnostic and prognostic values [25,46,47]. Similarly to TAPSE, the velocity of the longitudinal systolic contraction—S’ wave—determined at the lateral tricuspid annulus reflects the function of the longitudinal fibers [46,49,50]. The S’ wave is usually obtained from an apical 4-chamber view by placing the tissue Doppler cursor at the level of the tricuspid annulus or at the level of the basal segment of the RV free wall. Attention should be paid to the parallel alignment of the Doppler beam with the direction of RV longitudinal excursion. The advantages and disadvantages related to S’ wave are comparable to those observed when performing TAPSE [46,49,50]. It is simple to obtain, and has prognostic value, but it is angle dependent, influenced by the overall motion of the heart, and does not always accurately reflect the global RV systolic function [46]. The reference value for pulsed tissue Doppler S’ wave is 9.5 cm/s, a lower value predicting a LVEF < 40% [46,49,50].

Of all the estimation markers used in practice, RV fractional area change (RVFAC) correlates best with RV ejection fraction (RVEF) because the visible regions represent 80% of the RV, thus providing information on both longitudinal and radial contraction [25,46,47]. The limitation of this method is the uncertain boundary of the RV lateral wall when tracing the endocardial contour [46,49,50]. The normal RVFAC is 35–60%, and is an independent predictor of heart failure, sudden cardiac death, and postoperative outcome in cardiovascular surgery [46,49,50].

MPI provides information on global RV function (systolic-diastolic) and is independent of volumetric determinations as it is a dimensionless parameter (13). The RV myocardial performance index can be obtained using pulsed Doppler or tissue Doppler interrogation [46,49,50]. The advantage of MPI is that it depends only on time intervals, which makes it possible to avoid limitations related to the complex structure of the RV. However, MPI is task-dependent, being unreliable when right atrial (RA) pressure is elevated, the heart rhythm is irregular (atrial fibrillation) or there are conduction disturbances [46,49,50]. Given the complex anatomy of the RV, no single echocardiographic parameter is definitive [13]. A multimodal approach incorporating clinical scores, imaging findings, and biomarker levels provides the most accurate assessment of PE severity [13]. When used together, biomarkers and RV imaging enhance the identification of intermediate-risk patients who may benefit from closer monitoring or escalated therapy, as recommended by international guidelines [13]. To date, the combination of RV dysfunction identified via echocardiography or CTPA with a positive cardiac troponin test is the only strategy that has been prospectively evaluated in a large randomized controlled trial (RCT) to guide early therapeutic decision-making; specifically, the choice between anticoagulation alone and anticoagulation combined with reperfusion therapy in patients with PE who do not present hemodynamic instability [51].

Although echocardiographic assessment and the use of risk scores are commonly applied in current practice, they can sometimes be limited by the clinical context, which reiterates the usefulness and necessity of introducing biomarkers to facilitate more efficient patient assessment [52,53].

## 3. Biomarkers Correlated with Right Ventricular Dysfunction in Pulmonary Thromboembolism

The introduction of biomarkers has revolutionized the diagnosis and management of cardiovascular diseases, providing objective and quantifiable measures to assess cardiac stress, injury, and inflammation [54,55]. These biomarkers have been particularly instrumental in the diagnosis of acute coronary syndromes and heart failure, aiding both early detection and establishing prognosis, with troponins and natriuretic peptides serving as prime examples [10,11,12]. In the context of PE, biomarkers offer a non-invasive approach to assess RV strain and dysfunction, and inflammatory responses, enabling early identification of patients at risk of hemodynamic deterioration [56,57]. Among the most extensively studied biomarkers for RV dysfunction in PE are hs-cTnI, NT-proBNP, H-FABP, and GDF-15 [14,36,37]. These biomarkers not only provide insights into the underlying pathophysiological mechanisms of RV dysfunction, but also facilitate early detection, risk stratification, and prognostic evaluation [45,58]. When integrated into routine clinical assessments, these biomarkers can assist in optimizing patient monitoring strategies thereby enhancing the accuracy of risk assessment and management in intermediate-risk PE cases [57,59].

### 3.1. High-Sensitivity Cardiac Troponin I (hs-cTnI)

**Figure 2:**
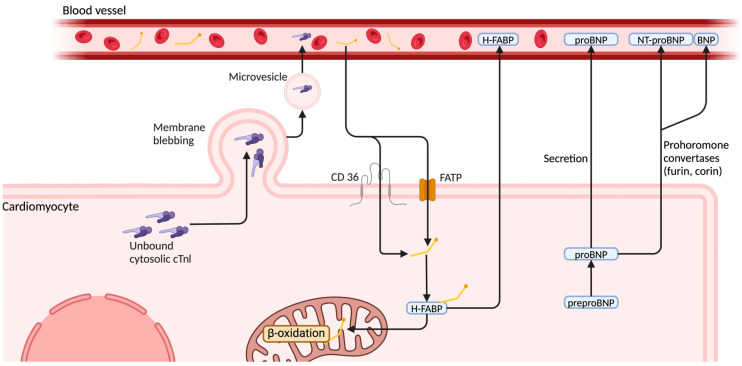
Pathways of biomarker secretion. NT-proBNP, N-terminal pro-B-type natriuretic peptide; H-FABP, heart-type fatty acid-binding protein; CD 36, cluster of differentiation 36; FATP, fatty acid transport protein. Created in BioRender (https://BioRender.com/l96f035, accessed on 16 April 2025).

High-sensitivity cardiac troponin I (hs-cTnI) is one of the most widely used biomarkers for detecting myocardial injury, with well-established clinical significance in the diagnosis of cardiovascular diseases [60,61]. Troponins are released into the bloodstream in response to cardiac myocyte injury, which may result from ischemia, mechanical stress, or other pathological processes [61]. This release forms the basis of their diagnostic utility, enabling clinicians to assess myocardial damage across various cardiovascular conditions [61]. Among troponin subtypes, hs-cTnI is particularly valuable due to its superior sensitivity, allowing for the detection of even minor myocardial injuries. This makes it especially relevant in conditions where myocardial damage may be subtle, or clinically overlooked such as RV strain [61,62]. In PE, hs-cTnI elevation primarily reflects myocardial stress and ischemia caused by acute RV pressure overload [16,57]. Structurally, cTnI is predominantly bound to myofilaments, with only a small unbound cytoplasmic fraction, accounting for approximately 3% of the total [35]. Under ischemic stress or membrane injury, this cytosolic fraction can be released into circulation through membrane blebbing (a form of membrane disruption), increased permeability, or packaging into microvesicles, independent of overt cell necrosis [63] (Figure 2). It is hypothesized that this cytoplasmic reserve is released secondary to myocardial injury induced by RV ischemia, which may explain the distinct troponin release pattern observed in PE compared to myocardial infarction (MI) [35]. In the context of PE, cTnI reaches its peak concentration approximately 10 h after symptom onset, and remains detectable for only 40 h [35,64]. The peak levels of cTnI in PE are lower than those observed in MI, and persist for a shorter duration [64]. Notably, in patients presenting more than 72 h after symptom onset, cTnI is often undetectable, despite the presence of RV dysfunction on echocardiography [35,64]. Understanding the kinetics of cTnI release is crucial for determining the optimal timing of blood sample collection to ensure accurate risk stratification [35,64]. The RV, in contrast to the LV, has a thinner wall and lower baseline pressures, rendering it more susceptible to sudden increases in afterload. When the pulmonary arteries are obstructed, RV afterload rises sharply, leading to mechanical stretching, ischemic injury, and subsequent troponin release [65,66,67]. Thus, this biomarker serves as an early indicator of myocardial damage that may not yet be clinically evident [65,66,67].

The pathophysiological significance of hs-cTnI elevation in PE lies in its association with RV ischemia, which occurs as a consequence of increased afterload and hemodynamic compromise [65,66,67]. Increased pulmonary vascular resistance reduces RV output, leading to systemic hypoperfusion and progressive cardiac dysfunction. Elevated hs-cTnI levels not only indicate acute myocardial stress, but also predict long-term complications such as chronic thromboembolic pulmonary hypertension (CTEPH) and recurrent thromboembolic events [65,66,67].

Evidence suggests that elevated hs-cTnI levels in PE patients correlate with increased mortality and adverse clinical outcomes [37,65,68]. Studies indicate that higher troponin levels are associated with an increased risk of heart failure, greater need for intensive medical intervention, and higher morbidity rates [2,69]. This prognostic value is particularly pronounced in intermediate- and high-risk PE patients, where RV dysfunction is more severe [70]. In these cases, hs-cTnI assists in risk stratification and guides therapeutic decision-making, including consideration for thrombolysis or catheter-directed interventions [71,72].

A study conducted by Choi et al. demonstrated that in a cohort of normotensive patients with acute PE, elevated cTnI levels (exceeding the 99th percentile of healthy individuals) measured at presentation or within 24 h of admission were associated with an increased risk of in-hospital or 30-day mortality [73]. Furthermore, elevated cTnI was linked to a higher need for cardiopulmonary resuscitation, vasopressor support, mechanical ventilation, and thrombolysis, establishing cTnI as a predictor of RV dysfunction [73]. Similarly, a study by Amorim et al. involving 77 patients diagnosed with PE found that among the 60 patients who underwent cTnI measurement, only 42 had elevated levels [74]. Among those with RV dysfunction, 26 (81.3%) exhibited increased cTnI levels, while only 14 (35%) of those with elevated cTnI did not present RV dysfunction. These findings suggest a significant association between elevated cTnI levels and RV dysfunction (*p* = 0.038) [74]. Additionally, cTnI levels were significantly higher in patients with submassive PE (mean level: 0.77 ng/mL) compared to those with non-massive PE (0.08 ng/mL, *p* < 0.05) [74]. The prognostic value of cTnI was further reinforced by Keller et al., who conducted a study on 182 patients with acute PE, of whom 129 met the inclusion criteria [65]. In this cohort, 45% of patients exhibited no echocardiographic evidence of RV dysfunction, whereas 55% met the criteria for RV dysfunction [65]. Notably, patients with RV dysfunction had significantly higher cTnI levels compared to those without dysfunction (0.06 ng/mL vs. 0.01 ng/mL, *p* < 0.0001) [65].

Despite its clinical utility, hs-cTnI is not specific to RV dysfunction, and may be elevated in a wide range of conditions, including acute coronary syndrome, myocarditis, sepsis, and systemic inflammatory responses [75]. This non-specificity can complicate its interpretation, particularly in critically ill patients where multiple pathophysiological mechanisms contribute to myocardial injury [76]. For instance, in sepsis, troponin elevation may result from cytokine-induced myocardial depression, rather than ischemic damage [75]. Similarly, in chronic kidney disease, impaired troponin clearance may lead to persistently elevated levels that do not necessarily indicate acute myocardial injury [75].

Given this complexity, hs-cTnI should be interpreted in conjunction with other clinical and diagnostic parameters. In PE, complementary tools such as echocardiography, CTPA, and additional biomarkers, such as BNP, provide valuable context for refining risk assessment and guiding treatment [77,78]. For instance, the presence of RV dilatation or dysfunction on echocardiography, when combined with elevated hs-cTnI levels, significantly strengthens the rationale for more aggressive therapeutic interventions [10].

In conclusion, hs-cTnI serves as a highly sensitive biomarker for myocardial injury and plays a critical role in risk stratification among PE patients [10]. Its elevation reflects ischemic stress within the RV due to increased afterload, making it a valuable tool for assessing disease severity and predicting adverse outcomes [10]. However, due to its lack of specificity, hs-cTnI should not be used in isolation, but rather in conjunction with clinical, imaging, and laboratory findings for a comprehensive evaluation [76]. By integrating troponin measurements with multimodal diagnostic approaches, clinicians can improve risk assessment and optimize therapeutic strategies, ultimately enhancing patient outcomes in PE and other cardiovascular conditions.

### 3.2. N-Terminal Pro-B-Type Natriuretic Peptide (NT-proBNP)

N-terminal pro-B-type natriuretic peptide (NT-proBNP) is a well-established biomarker of myocardial stress and ventricular strain, particularly in conditions characterized by increased cardiac wall tension, such as heart failure, PE, and RV dysfunction [79]. NT-proBNP is synthesized in response to mechanical stretching of myocardial fibers, which occurs when the heart is subjected to excessive volume or pressure overload [79]. Upon release, proBNP, the precursor peptide, is cleaved into two components by prohormone convertases (furin and corin) into biologically active BNP and inactive NT-proBNP [67,80] (Figure 2). Since NT-proBNP secretion is directly proportional to myocardial fiber stretching, it serves as a valuable marker for assessing ventricular strain, particularly in PE, where RV overload is a significant clinical concern [81]. Unlike troponin, which is a structural component of cardiomyocytes, only small amounts of BNP and NT-proBNP are stored within the cell under physiological conditions [67]. Instead, their secretion is stimulated by a constitutive mechanism in response to myocardial stretching, resulting detectable circulating levels after several hours [67]. In PE, NT-proBNP levels rise due to acute RV afterload caused by pulmonary arterial obstruction. As the thrombus increases resistance within the pulmonary circulation, the RV must generate higher pressures to maintain blood flow, leading to excessive myocardial stretching and subsequent NT-proBNP release [82,83]. Unlike the LV, the RV is structurally less equipped to withstand sustained pressure overload, making it highly vulnerable to dysfunction, ischemia and, in severe cases, failure [67,83].

Although NT-proBNP is widely used in the diagnosis and management of heart failure, its role in assessing RV dysfunction in PE has only recently gained increased recognition [84]. In the context of PE, NT-proBNP provides important prognostic insights regarding the degree of RV overload and its potential clinical consequences [85]. Numerous studies have shown that elevated NT-proBNP levels are associated with increased mortality and a higher likelihood of requiring intensive care interventions [85].

A commonly recognized threshold for significant RV dysfunction in PE is an NT-proBNP level exceeding 600 pg/mL [86]. Patients surpassing this threshold face a significantly higher risk of complications, including hemodynamic collapse, cardiogenic shock, and death [85]. Moreover, progressive elevations in NT-proBNP have been linked to worsening prognosis, emphasizing the biomarker’s utility in early risk stratification [39,85]. A meta-analysis of 1132 patients with acute PE demonstrated that increased BNP and NT-proBNP concentrations correlated with a higher risk of 30-day mortality and severe complications, including cardiopulmonary resuscitation, mechanical ventilation, vasopressor use, thrombolysis, thrombectomy, or intensive care unit admission [55].

In the study of Cotugno et al., 52 out of 172 patients with echocardiographically confirmed RV dysfunction exhibited significantly higher mean NT-proBNP levels compared to those without dysfunction [67]. Similarly, Pruszczyk et al. demonstrated that elevated NT-proBNP levels were strongly associated with the presence of RV dysfunction on echocardiography. In their study, patients with RV dysfunction had significantly higher NT-proBNP levels (4650 pg/mL) compared to those without dysfunction (363 pg/mL) [87].

Although NT-proBNP is a robust marker of cardiovascular stress, it is not exclusive to PE or RV dysfunction [82]. Similarly to hs-cTnI, NT-proBNP levels may be elevated in conditions such as heart failure, chronic kidney disease, systemic infections, and acute coronary syndromes [88]. This lack of specificity underscores the necessity of interpreting NT-proBNP levels within a broader clinical and diagnostic framework, rather than in isolation [88].

Despite its limitations, NT-proBNP remains an invaluable tool in the evaluation of PE, contributing to early diagnosis, risk stratification, and prognostic assessment [89]. When combined with imaging modalities such as echocardiography and CTPA, NT-proBNP measurement enhances diagnostic precision, facilitating the identification of high-risk patients who may benefit from advanced therapeutic interventions, including thrombolysis or catheter-directed therapy [89].

### 3.3. Heart-Type Fatty Acid-Binding Protein (H-FABP)

Heart-type fatty acid-binding protein (H-FABP) is a cytoplasmic protein primarily expressed in cells with a high fatty acid metabolism, such as cardiomyocytes. It has gained recognition as a novel and sensitive biomarker for detecting myocardial injury, ischemia, and hypoxia [54]. Unlike cardiac troponins, which are predominantly bound to the contractile elements of cardiomyocytes and released upon myocardial cell necrosis, H-FABP is upregulated and released before cell death occurs, making it a more sensitive marker for early myocardial stress [53,90]. H-FABP—a small cytoplasmatic protein involved in fatty acid transport to mitochondria—diffuses rapidly through the interstitial space and, due to its small molecular size (15 kDa), appears in circulation within 90–120 min from cardiac injury [35,91,92]. H-FABP trafficking is mediated by fatty acid transporters, including cluster of differentiation 36 (CD36) and fatty acid transport proteins (FATP), and contributes to β-oxidation under normal conditions [92]. It reaches peak plasma concentration at 6–8 h, and returns to baseline within 24–36 h due to rapid renal clearance [35,91]. This kinetic characteristic is particularly advantageous in conditions such as PE, where RV strain and ischemia precede overt myocardial damage and timely intervention is crucial for improving clinical outcomes [93]. In the context of PE, H-FABP can detect subclinical myocardial injury at a stage where conventional biomarkers may remain undetectable, making it an important tool for early risk stratification [54].

The role of H-FABP in PE was first demonstrated by Kaczynska et al. in 2006, in a prospective cohort study of 77 patients, including 9 with massive PE, 43 with submassive PE, and 25 with non-massive PE [94]. When compared to cTn, NT-proBNP, and myoglobin, H-FABP was the only biomarker that predicted both 30-day PE-related mortality and all-cause mortality [94].

A growing body of evidence suggests that elevated H-FABP levels in PE patients correlate with poorer clinical outcomes, including higher rates of complications and lower survival rates [87]. Similar findings were reported by Dellas et al. in a study on 126 hemodynamically stable patients with acute PE [95]. H-FABP levels at presentation ranged from 0.39 to 217.5 ng/mL, with a median value of 3.4 ng/mL [95]. Patients who developed complications had significantly higher median H-FABP levels at admission compared to those with an uncomplicated clinical course, a difference that was statistically significant (*p* < 0.001) [95]. Additionally, patients with elevated H-FABP levels tended to be older, exhibited higher plasma concentrations of cTn and NT-proBNP, and were more likely to present with echocardiographic evidence of RV dysfunction [95]. The study further demonstrated that elevated H-FABP levels were predictive of both short-term adverse outcomes and long-term mortality in patients with intermediate-risk PE [95]. Notably, patients with negative H-FABP levels at admission remained negative within the first 24 h, suggesting that repeated biomarker measurements may not be necessary to enhance its prognostic value [95]. Consequently, integrating H-FABP into risk assessment protocols for PE patients may enhance clinical decision-making and optimize resource allocation. Furthermore, H-FABP demonstrates higher specificity for myocardial ischemia compared to other cardiac biomarkers, such as BNP or D-dimers, which may be elevated in various non-cardiac conditions [96]. Moreover, H-FABP levels are associated with worse clinical outcomes, even when troponins and BNP levels remain within normal ranges [96]. A meta-analysis of nine studies including 1680 patients further reinforced the prognostic utility of H-FABP in PE. Elevated H-FABP levels were strongly associated with an increased risk of RV dysfunction, adverse clinical outcomes, and 30-day mortality [54,97]. When compared with hs-cTn, BNP, and NT-proBNP, H-FABP emerged as the strongest predictor of both short-term PE-related mortality and all-cause mortality [54,97]. The incorporation of H-FABP into existing risk models for PE could improve clinical decision-making by identifying high-risk patients earlier, reducing unnecessary hospital admissions in low-risk patients, optimizing healthcare resources, and enhancing survival rates by enabling earlier and more targeted therapeutic interventions.

### 3.4. Growth Differentiation Factor 15 (GDF-15)

Growth differentiation factor-15 (GDF-15) is a member of the transforming growth factor-beta (TGF-β) superfamily of cytokines, known for its role in cellular growth, differentiation, and stress response. While GDF-15 is not typically expressed in myocardial tissue under normal physiological conditions, its production increases in response to pressure overload, ischemia, and oxidative stress, making it a valuable prognostic biomarker, particularly in acute coronary syndromes and chronic heart failure [98]. Based on these findings, it was hypothesized that GDF-15 could provide important prognostic insights into RV dysfunction, particularly in patients with PE, including higher mortality rates and more severe RV dysfunction [98]. Being a stress-responsive cytokine that is upregulated in response to inflammatory and oxidative stress, GDF-15 was associated with protective mechanisms against ischemia and apoptosis, particularly through pathways mediated by angiotensin II, nitric oxide (NO), and TGF-β1 [99]. Its effects on myocardial remodeling remain complex, as both pro-hypertrophic and anti-hypertrophic properties have been described [99].

In ischemic models, GDF-15 exerts anti-apoptotic effects by activating Small mother against decapentaplegic (Smad1) signaling, which leads to upregulation of B-cell lymphoma-extra large (Bcl-xL) and β-catenin, thereby reducing myocardial infarct size [100]. GDF-15 shares some structural and signaling characteristics with bone morphogenetic family proteins (BMPs), particularly BMP-2. GDF-15 acts through a broad range of receptors and pathways, including Smad-independent mediators like mitogen-activated protein kinases (MAPKs), transforming growth factor-β-activated kinase 1 (TAK1), and phosphatidylinositol 3-kinase/protein kinase B (PI3K/Akt), depending on the specific cellular type of injury [101]. Beyond Smad pathways, GDF-15 exerts protective effects via alternative signaling routes. Xu et al. showed that in models of norepinephrine (NE)-induced cardiac hypertrophy, GDF-15 counteracts pathological growth responses not by Smad activation, but by interfering with epidermal growth factor receptor (EGFR) transactivation, a known downstream effect of G protein-coupled receptor (GPCR) signaling [102]. NE stimulation typically leads to EGFR activation via intermediary mechanisms, such as metalloproteinase-mediated release of EGF ligands [102]. GDF-15 inhibits this EGFR transactivation, resulting in reduced phosphorylation of key hypertrophic mediators, such as Akt and extracellular signal-regulated kinase (ERK), thereby mitigating the hypertrophic response [102]. In endothelial cells, GDF-15 provides resistance against oxidative and inflammatory injury. Li et al. investigated human umbilical vein endothelial cells (HUVECs) exposed to high glucose as a model of metabolic stress [103]. Their findings revealed that silencing GDF-15 enhanced apoptosis via activation of the NF-κB/JNK/caspase-3 axis, a canonical inflammatory pathway known to induce apoptosis [103]. Conversely, exogenous GDF-15 restored cellular homeostasis by suppressing this pro-apoptotic cascade and simultaneously activating the PI3K/Akt/eNOS pathway, promoting NO production and enhancing cell survival [103]. The reduction in NO observed in GDF-15-deficient cells further supported the importance of this pathway in endothelial function [103].

**Figure 3:**
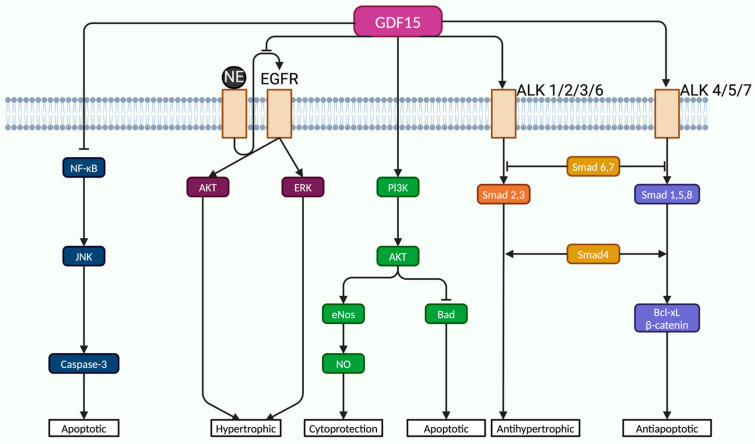
GDF-15 signaling pathways. NF-κB, nuclear factor kappa-light-chain-enhancer of activated B cells; JNK, Jun N-terminal kinase; ERK, extracellular signal-regulated kinase; PI3K, phosphatidylinositol-3 kinase; EGFR, epidermal growth factor receptor. Created in BioRender (https://BioRender.com/l96f035, accessed on 16 April 2025).

Overall, GDF-15 supports cardiovascular protection through several well-established pathways. It helps reduce inflammation and prevent cell death by inhibiting the NF-κB/Jun N-terminal kinase (JNK)/caspase-3 signaling cascade while also enhancing endothelial cell survival by activating the PI3K/Akt/eNOS pathway [103]. In the context of cardiac hypertrophy, GDF-15 works through both Smad-dependent signaling (like Smad2/3) and alternative, non-Smad routes such as MAPK and TAK1 pathways [101,102]. Additionally, it interferes with harmful signaling between GPCRs and EGFR, thereby limiting downstream Akt and ERK activation that would otherwise promote pathological cell growth [102] (Figure 3).

The first study to report elevated GDF-15 levels in patients with PE was conducted by Lankeit et al. in 2008 [104]. They demonstrated that patients with increased GDF-15 concentrations at hospital admission had nearly a threefold higher risk of mortality compared to those with normal levels [104]. Plasma GDF-15 was measured in 123 patients with acute PE, with an upper limit of 1200 ng/L defined as the threshold [104]. Although patients with elevated GDF-15 levels tended to be older and more frequently in cardiogenic shock, or with comorbidities such as chronic heart failure, diabetes, malignancy, or renal dysfunction, none of the patients with GDF-15 levels below 1200 ng/L experienced major complications within the first 30 days [104]. Further analysis from this study established a prognostic cutoff of 4600 ng/L, which was associated with a sensitivity of 0.71, a specificity of 0.90, and a negative predictive value of 0.95 for adverse outcomes [104]. Moreover, initial GDF-15 levels were identified as independent predictors of long-term mortality (*p* < 0.001) [104]. However, due to the non-specific nature of GDF-15 as a biomarker for myocardial injury, its elevation in PE cannot be solely attributed to acute RV overload. Therefore, GDF-15 measurements should be considered alongside other biomarkers, such as NT-proBNP and cardiac troponins, to improve diagnostic accuracy [96,105,106]. Its role in predicting long-term outcomes is of particular interest, as persistent RV dysfunction following PE has been associated with CTEPH and heart failure. Early identification of patients with high GDF-15 levels could therefore facilitate closer monitoring and tailored treatment strategies [96,105,106].

The prognostic significance of GDF-15 in PE was further supported by Duran et al., who demonstrated that GDF-15 was superior to NT-proBNP in predicting early mortality in normotensive PE patients [107]. Although GDF-15, cTnT, and NT-proBNP exhibited similar sensitivities for predicting severe adverse events, GDF-15 emerged as a key predictor of early hemodynamic instability and hemorrhagic complications in PE [39]. However, while GDF-15 offers promise as a biomarker for PE severity assessment, it has not yet been integrated into routine clinical practice due to several challenges, including the lack of standardized cutoff values and limited validation in large-scale studies [53].

Although GDF-15 is not yet widely implemented in clinical practice, ongoing research suggests that it may become a pivotal biomarker for both severity assessment in RV dysfunction and prediction of long-term outcomes in PE patients. If future studies establish clear prognostic thresholds, standardized testing protocols, and clinical guidelines for its use, GDF-15 could significantly enhance risk stratification strategies in acute PE management.

### 3.5. Clinical Utility of Biomarkers Across Risk Stratification Subgroups in PE

Although biomarkers such as hs-cTnI, NT-proBNP, H-FABP, and GDF-15 are widely studied in PE, their clinical relevance must be interpreted within the context of already-established risk categories (Table 1). Biomarkers alone are not sufficient for risk assessment. However, their value lies in serving as adjuncts to clinical and imaging findings [108]. Their clinical utility vary significantly across the three major risk subgroups, defined by current guidelines based on hemodynamic status and RV imaging findings [13]. Patients with systolic blood pressure < 90 mmHg (hemodynamically unstable) or in shock have high-risk PE. In these cases, the presence of RV dysfunction is assumed, and biomarkers have limited value, as they do not influence the immediate clinical decision-making process [13]. These patients require immediate reperfusion therapy, either systemic thrombolysis or catheter-directed intervention. Thus, biomarkers are not clinically useful, as treatment decisions are based on hemodynamic criteria. Intermediate-risk PE patients benefits most from biomarker use. Patients who are normotensive but have evidence of RV dysfunction on echocardiography or CTPA should undergo further risk stratification [13,44]. Biomarkers, especially troponins and NT-proBNP, can help identify those at intermediate-high risk, who are at high risk of hemodynamic deterioration, and guide decisions about ICU admission or closer monitoring [13,45].

Studies comparing BNP and troponin suggest that they have complementary value, as BNP reflects RV strain, while troponins reflect ischemic injury. Strong supporting evidence was provided by Kucher et al.’s study that enrolled normotensive patients with acute PE. While BNP levels > 90 pg/mL had high sensitivity but relatively low specificity for RV dysfunction [109], elevated troponin T levels (>0.01 ng/mL) demonstrated lower sensitivity but higher specificity for adverse outcomes [109]. Importantly, the study found that patients with concurrent elevation of both BNP and troponin T faced significantly higher risk of complications, emphasizing the value of dual-marker assessment [109]. While BNP offers higher sensitivity for detecting RV dysfunction, troponins, especially hs-cTnT, are more specific for adverse outcomes [110]. Lankeit et al. study demonstrated superior prognostic sensitivity and negative predictive value of hs-cTnT over conventional assays. Furthermore, hs-cTnT effectively predicted both early complications and long-term mortality [111]. The use of both biomarkers improves risk stratification in normotensive PE patients, particularly facilitating the identification of those at intermediate-high-risk who require closer monitoring or escalated care. When used together, they may enhance prognostic accuracy.

Notably, the PEITHO trial demonstrated that intermediate-risk PE patients with both RV dysfunction and positive cardiac biomarkers were more likely to benefit from closer monitoring or advanced therapy [112]. Even in patients with normal vital signs and no RV dysfunction on imaging, classified as low-risk PE, biomarkers may detect early myocardial stress or subclinical RV injury [13,45]. Recent evidence shows that pro-BNP has a significantly better diagnostic accuracy than troponin I [113]. At a cut-off of 100 pg/mL, pro-BNP has a sensitivity of 85.4% and specificity of 80.2% [113]. In contrast, troponin I had lower diagnostic value [113]. Similarly, a retrospective multicenter study comparing the prognostic value of BNP and cTn in predicting in-hospital mortality in a cohort of 758 patients with acute PE reinforced previous findings. BNP elevation showed higher sensitivity (81.2%) and negative predictive value (96.8%) than troponin [114]. Therefore, pro-BNP has clinical utility as sensitive and specific marker for early diagnosis and risk stratification in PE, particularly when used alongside imaging and other laboratory tests.

Elevated NT-proBNP or H-FABP levels have been associated with worse short-term outcomes, suggesting that biomarker testing may uncover hidden risk and may help identify those patients in the low-risk category who require closer follow-up or inpatient observation [38,115,116]. In selected cases, biomarkers may help refine prognosis even when other assessments indicate low risk.

## 4. Conclusions

Attempts to find new methods to correlate congestive heart failure with right ventricular dysfunction, especially in the setting of pulmonary embolism, have revealed new biomarkers with a promising potential prognostic role. Although the few studies currently available lack the power to support validation in current practice, they have consistently shown that hs-cTnI and NT-proBNP are strong predictors of mortality in patients with intermediate- and high-risk PE, as they reflect myocardial injury, significant RV strain, and severity of RV overload. H-FABP is emerging as a promising early predictive biomarker of myocardial injury, especially for early diagnosis of RV strain before the occurrence of clinically significant myocardial necrosis. Furthermore, GDF-15 has been identified as an important biomarker in long-term risk prediction, increased levels correlating with worse outcomes and RV remodeling. Nevertheless, future research should focus on developing clinical algorithms that incorporate synergistic combination of biomarkers, together with imaging techniques such as echocardiography and CTPA, to improve the overall assessment of RV dysfunction in PE. Challenges remain in the widespread use of these biomarkers, including their associated high cost, and limited accessibility in some clinical settings. Further research is required to define standard thresholds, improve cost-effectiveness, and clarify their role in the management of PE-associated RV dysfunction.

## Figures and Tables

**Table 1:** Comparison of biomarkers.

Refs.	Biomarker	Mechanism	Kinetics	Clinical Utility	Limitations
[35,37,57,65,68,73,74]	Hs-cTnI	myocardial injury due to RV ischemia from pressure overload	- peaks in ~10 h - detectable for ~40 h	- early marker of myocardial stress - correlates with RV dysfunction severity - predicts mortality and complications	- low specificity in PE or RV injury - elevated in ACS, sepsis, CKD
[55,67,81,85,86,87,89]	NT-proBNP	ventricular wall stretch from acute RV pressure overload	- increases within hours - persists longer than troponin	- strong prognostic marker - useful threshold: >600 pg/mL - predicts ICU need and 30-day mortality	- elevated in heart failure, renal dysfunction
[54,94,95,97]	H-FABP	early release due to ischemia and cardiomyocyte stress (before necrosis)	- appears in 1.5–2 h - peaks in 6–8 h - clears in 24–36 h	- sensitive to early RV ischemia - strong predictor of short-term mortality - higher specificity for ischemia than D-dimer or BNP in some cases	- short half-life - less established in clinical use
[39,96,98,104,107]	GDF-15	stress-induced cytokine upregulated in response to pressure, ischemia, inflammation	- elevates under sustained cardiac or systemic stress	- predicts early- and long-term mortality - high NPV at thresholds <1200 ng/L - useful in normotensive PE	- non-specific; - elevated in cancer, CKD, HF - not yet standard in clinical algorithms

ACS, acute coronary syndrome; CKD, chronic kidney disease; ICU, intensive care unit; NPV, negative predictive value.

## Data Availability

No new data were created or analyzed in this study. Data sharing is not applicable to this article.
